# Center-environment feature models for materials image segmentation based on machine learning

**DOI:** 10.1038/s41598-022-16824-w

**Published:** 2022-07-28

**Authors:** Yuexing Han, Ruiqi Li, Shen Yang, Qiaochuan Chen, Bing Wang, Yi Liu

**Affiliations:** 1 School of Computer Engineering and Science, Shanghai University, 99 Shangda Road, Shanghai, 200444 China; 2 Zhejiang Laboratory, 311100 Hangzhou, China; 3 Materials Genome Institute, Shanghai University, 333 Nanchen Road, Shanghai, 200444 China

**Keywords:** Computer science, Theory and computation

## Abstract

Materials properties depend not only on their compositions but also their microstructures under various processing conditions. So far, the analyses of complex microstructure images rely mostly on human experience, lack of automatic quantitative characterization methods. Machine learning provides an emerging vital tool to identify various complex materials phases in an intelligent manner. In this work, we propose a “center-environment segmentation” (CES) feature model for image segmentation based on machine learning method with environment features and the annotation input of domain knowledge. The CES model introduces the information of neighbourhood as the features of a given pixel, reflecting the relationships between the studied pixel and its surrounding environment. Then, an iterative integrated machine learning method is adopted to train and correct the image segmentation model. The CES model was successfully applied to segment seven different material images with complex texture ranging from steels to woods. The overall performance of the CES method in determining boundary contours is better than many conventional methods in the case study of the segmentation of steel image. This work shows that the iterative introduction of domain knowledge and environment features improve the accuracy of machine learning based image segmentation for various complex materials microstructures.

## Introduction

With the rapid development of science and technology, technological innovation in industrial fields cannot be separated from the development of new materials. However, developing new materials and mass production require a long research cycle, which fails to meet the requirements of the industry for the speed of research and development of new materials. Thus, how to shorten the material development cycle as much as possible has become one of major topics. Many countries around the world have attached great importance to the funding and policy support in this field until now.

In the process of researching and developing new materials, studying relationships among macroscopic properties, microstructure and preparation technology is a key step^1^. The abundant microstructural information in material images, e.g., boundaries, textures, distributions, are highly relevant to materials properties^2^. Material phase recognition is one of the most important targets, which can quantitatively help to analyze the properties of materials. However, there are some challenges for material image processing as follows: first, most material images contain a large number of fuzzy boundaries and complicated irregular textures, as shown in Figure S1; second, the different material images show various types of textures, so substantial expert domain knowledge is needed for image annotation, recognition, and segmentation; third, the number of consistent and classified material samples is too small due to the cost of sample preparation and characterization.

The methods to solve the problem of image segmentation and recognition are as follows: 1. domain knowledge of experts and rule methods; 2. statistical models and machine learning methods; 3. automatic feature extraction and recognition based on deep neural network methods. Most non-deep learning methods for image segmentation and recognition are automatically executed by some special algorithms. The effect of results mainly depends on domain knowledge based annotation and precision of methods, and the results cannot be changed^3,4^. Although the non-deep learning methods perform well on the material images with the prescribed features, they are not applicable for material images with unknown configurations because these textures exhibit various morphology. Many non-deep learning methods do not make full use of pixel’s environment information. Thus, they are difficult to distinguish textures in areas with similar local pixels. Obviously, some deep learning methods can solve the problem of complicated texture segmentation, but they require large scale training samples and extra accurate manual annotations^5,6^. While most kinds of material images are small samples and are unable to be annotated accurately. Therefore, the deep learning methods encounter difficulty in material image processing.

To overcome the above mentioned shortages, an interactive image segmentation is proposed here based on extracting center-environment features of each pixel. Our method considers domain knowledge based center-environment features for the small samples of material images. The center-environment features consist of domain knowledge based spatial information and several common texture features that can better represent rich and complex texture information in pixels and their adjacent pixels. Annotators can iteratively draw several curves in each region based on experts’ domain knowledge. Pixels on curves and their adjacent pixels are fed into the feature extractor simultaneously. Since we annotate each category by drawing curves on different textures, the numbers of the different categories of pixels are similar. For classifier, we choose some machine learning algorithms to compare the intersection over Union (IoU), mean intersection over Union (mIoU), Accuracy, Dice Coefficient and mDice (mean Dice Coefficient) on many types of material images. The comparing results show that GBDT (Gradient Boosting Decision Tree)^7^ performs the best.

We evaluate our method on multiple material data sets. The experiment process is shown on carbon steel micrograph data set in details. Furthermore, we also test our method with different types of material image to show our method has a certain range of application. The experimental tests demonstrate that more accuracy boundary can be achieved on multiple data sets with our method under equivalent annotation cost. Furthermore, the proposed method only needs a few image data with limited annotation to train a segmentation model, where annotators draw curves repeatedly in the images to extract pixels’ center-environment features to improve the model in per round dynamically based on experts’ domain knowledge. Moreover, our method can work well with small number of samples of material images.

## Results

### Segmentation for images of carbon steel data set

#### Introduction of carbon steel data set

The carbon steel data sets are built using the UHCSDB (UltraHigh Carbon Steel Micrograph DataBase)^8^ including multiple phases. To analyze the features of the selected phases, i.e., the blue area in the annotation, segmenting the specific phase from the other phases in the images is a fundamental task.

#### Training and segmentation

**Figure 1:**
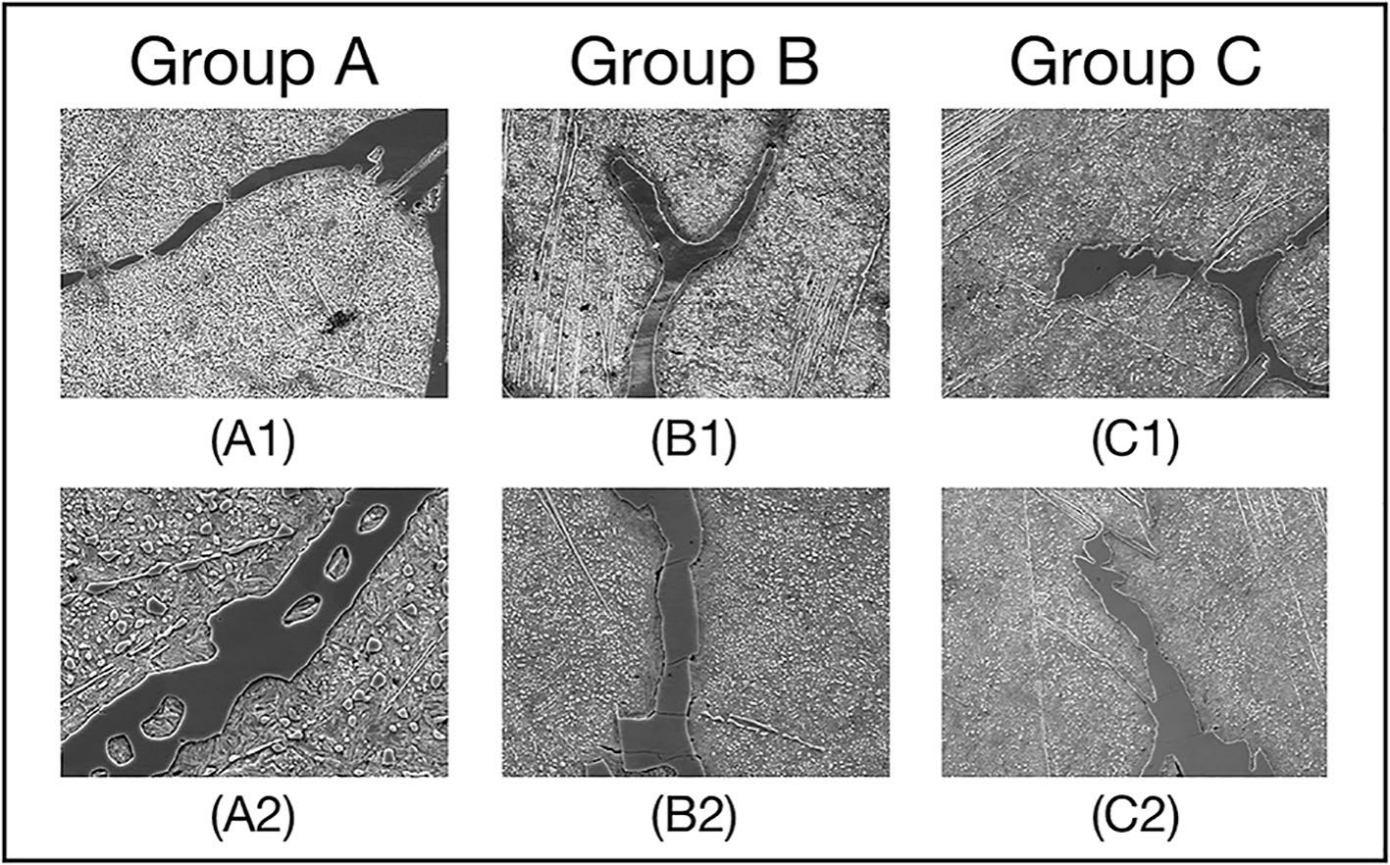
Division of data set in carbon steel images. Each group has two images. The primary microconstituent of (**A1**,**A2**,**B2**,**C1**,**C2** )is spheroidite, and the primary microconstituent of (**B1**) is spheroidite and widmanstatten.

**Figure 2:**
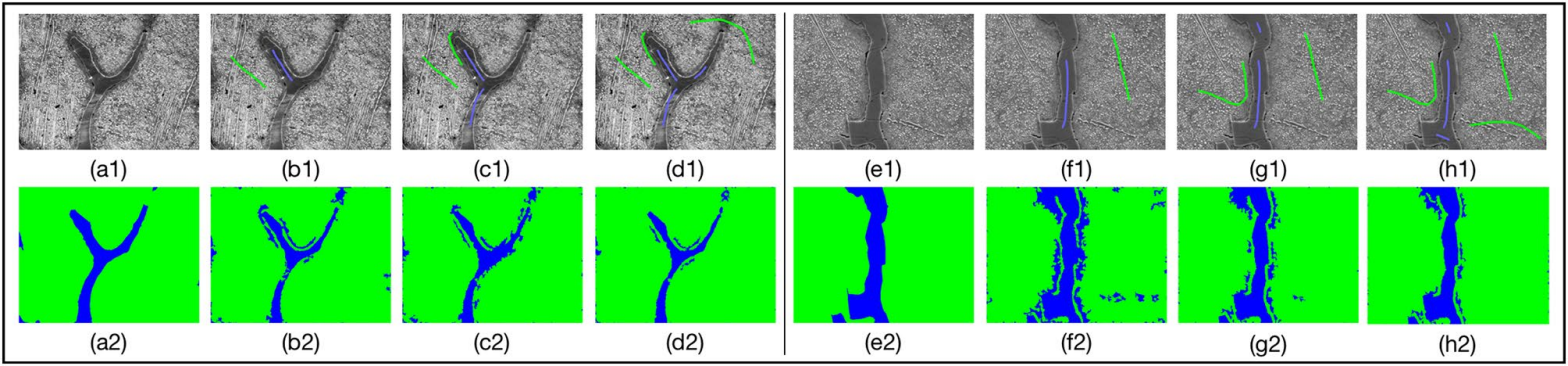
Training process of images in group B. (**a1**,**e1**) are the original images of group B. (**a2**,**e2**) are the ground truth. (**b1**,**c1**,**d1**), (**f1**,**g1**,**h1**) are the training processes for (**a1**) and (**e1**), respectively. (**b2**,**c2**,**d2**,**f2**,**g2**,**h2**) are their results corresponding with per training round.

**Figure 3:**
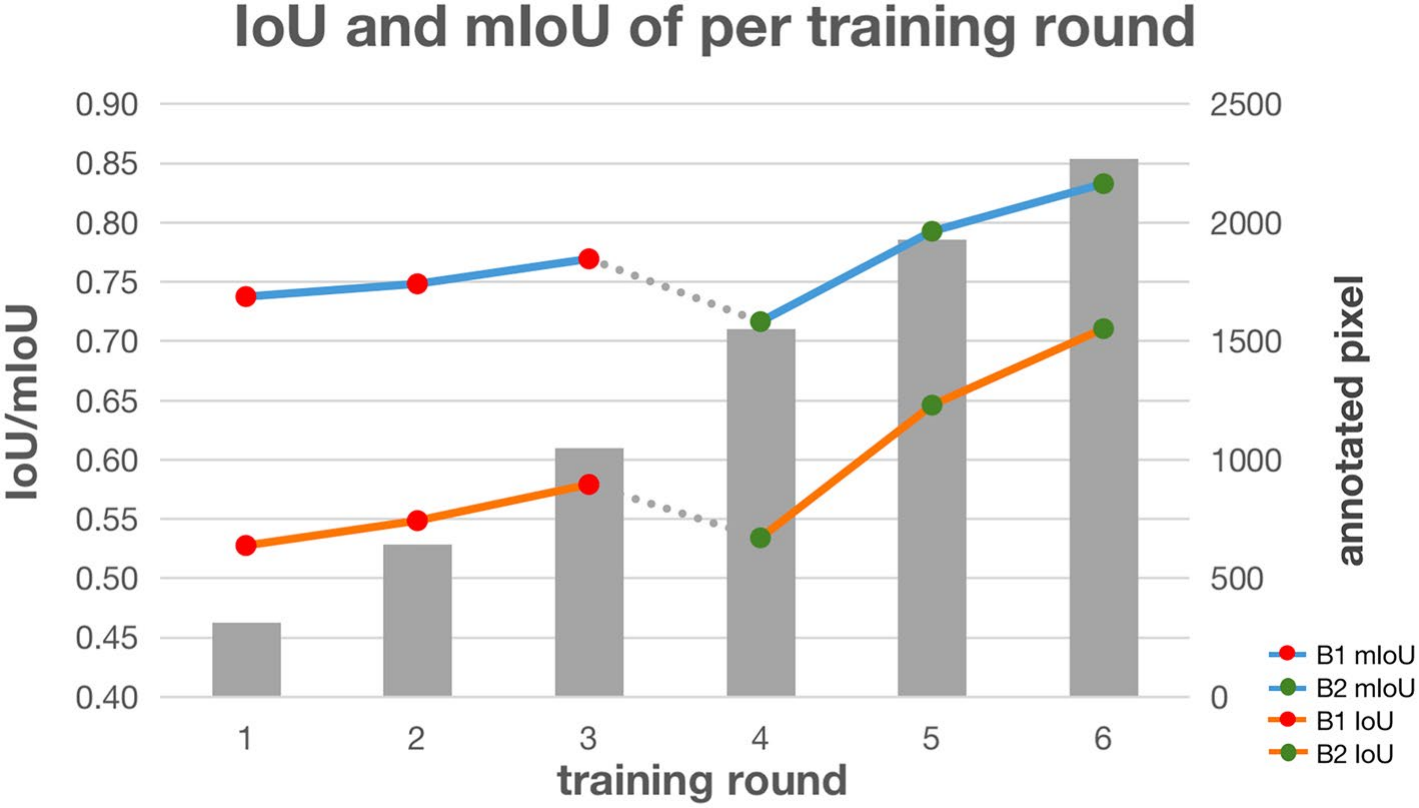
The IoU, mIoU and annoteted pixels of group B after 6 training rounds. The gray bar indicates the cumulative number of annotated pixels, i.e., the annotation cost.

To obtain the region of interest (ROI) of each pixel, we extend each edge of the image by 4 pixels. The value of expanded pixels are set as 0. Subsequently, we obtain the square region around each pixel one by one as the ROI of the current pixel. The side length of ROI is not a fixed value. If the side length of ROI is large, the area used to describe the texture is large. It can improve the segmentation accuracy, but the phase boundaries may be blurred. On the other hand, if the side length of ROI is small, the extracted features are less accurate for classification, but the feature extraction is faster and the phase boundaries is not blurred. We confirm size of region of interest (ROI), i.e., center-environment scope, as 9 by experiments. As shown in Fig. 1, we divide the 6 images from the UHCSDB into 3 groups for cross validation. In training process and segmentation process, the scale bars are cropped first. Our experiment applies the models generated by training the 3 groups of Fig. 1, respectively, on the other two groups. The training processes of 3 groups are shown in Fig. 2, S2 and S3, respectively. And the segmentation results are shown in Figure S4, S5, S6. Figure 2 shows the training process of images in group B. Each training image is iteratively annotated and trained for 3 times. In the first round, two marked curves in two colors represent two types of phases. The pixels covered on the marked curves and the adjacent pixels in ROI are all fed into feature extractor to generate feature vectors. The segmentation results of first round show that some pixels are incorrectly predicted, which needs to be further rectified. The follow-up rounds focus on the errors in the segmentation results of current round respectively, and the results show that the correction is obvious. We use common evaluation for segmentation, intersection over union (IoU), mean intersection over union (mIoU), Accuracy, Dice Coefficient (Dice) and mean Dice Coefficient (mDice), to evaluate results. After six rounds, the segmentation’s mIoU reaches 0.833. With more training rounds, the increase of annotation cost tends to surpass the increase of segmentation accuracy, shown as the gray bars in Fig. 3. Therefore, we choose six training rounds for the carbon steel images. Figure 3 shows the IoU, mIoU and cumulative annotated pixels of the 6 training rounds on B1 and B2 in Fig. 1. The results demonstrate that performance of segmentation is improved overall after multiple training rounds. The final average mIoU of training group B is over 0.8. The training and testing process of the other 2 groups are shown in Figure S2, S3, S4, S5 and Table S1.

**Figure 4:**
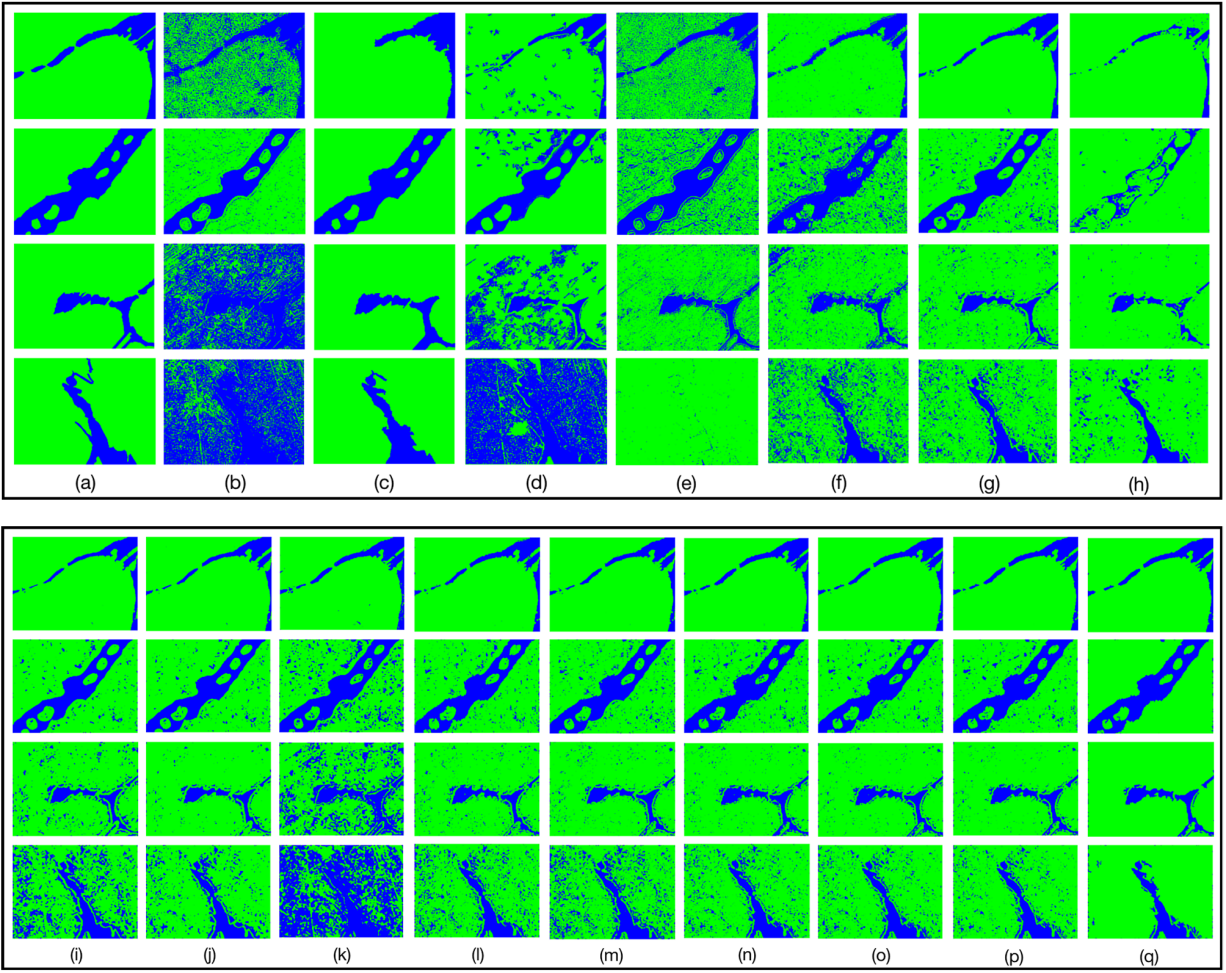
Comparison between our CES method with other methods. (**a**) ground truth; (**b**) MRF algorithm; (**c**) watershed algorithm; (**d**) Han’s algorithm; (**e**) meanshift algorithm; (**f**) DT; (**g**) KNN; (**h**) K-means; (**i**) Naive Bayes; (**j**) SVM (RBF); (**k**) SVM (Sigmoid); (**l**) RF; (**m**) AdaBoost; (**n**) XGBoost; (**o**) DART; (**p**) GBDT (our classifier); (**q**) CES (GBDT and post-processing process).

The six images of the carbon steel in Fig. 1 are randomly divided to 3 groups, and segmentation results are shown in Table S1. In Table S1, the images in group A and group C are better segmented with the model trained on group B, while the models trained on group A and C are less effective to segment the images in group B. It indicates that the information contained in the images of group B is more complex. Therefore, we use the images of group B to train our method and other methods to verify the effectiveness of our method. The compared methods include Markov Random Field algorithm (MRF)^9^, watershed algorithm^10^, Han’s algorithm^11^, meanshift algorithm^12^, decision tree (DT)^13^, K Near Neighbor (KNN)^14^, naive bayes^15^, K-means^16^, support vector machine (SVM)^17^ with different kernel functions, random forest (RF)^18^, Adaptive Boosting (AdaBoost)^19^, extreme Gradient Boosting (XGBoost)^20^, Dropouts meet multiple additive regression trees (DART)^21^ and Gradient Boosting Decision Tree (GBDT)^7^. Figure 4 shows the compared results. Due to the fuzzy boundaries, most other methods are difficult to predict the boundaries accurately. Our method can more accurately segment the material images, since feature vectors extracted from feature extractor can distinguish pixels well and the classifier has good learning ability. Table 1, S2, S3 and S4 show the IoU, mIoU, Accuracy, Dice and mDice of the 4 images based on the compared methods, which verifies that our method performs better than the most of these methods for distinguishing each region.

**Table 1:** The IoU (blue area), mIoU, Accuracy, Dice (blue area) and mDice of Image A1 using other methods and our CES method trained on group B.

Methods	IoU	mIoU	Accuracy	Dice	mDice
A1					
MRF	0.315	0.502	0.727	0.479	0.647
Watershed	0.763	0.862	0.966	0.866	0.923
Han	0.529	0.721	0.921	0.692	0.823
Meanshift	0.424	0.621	0.840	0.595	0.748
DT	0.708	0.831	0.958	0.829	0.903
KNN	0.688	0.821	0.958	0.815	0.896
K-means	0.352	0.631	0.914	0.521	0.737
Navie Bayes	0.664	0.807	0.955	0.798	0.886
SVM(RBF)	0.686	0.820	0.958	0.813	0.895
SVM(Sigmoid)	0.798	0.883	0.972	0.887	0.936
RF	0.723	0.841	0.963	0.840	0.909
AdaBoost	0.733	0.847	0.964	0.846	0.913
XGBoost	0.781	0.874	0.971	0.877	0.930
DART	0.769	0.867	0.969	0.870	0.926
GBDT	0.783	0.876	0.971	0.879	0.931
CES	0.788	0.878	0.972	0.881	0.933

**Figure 5:**
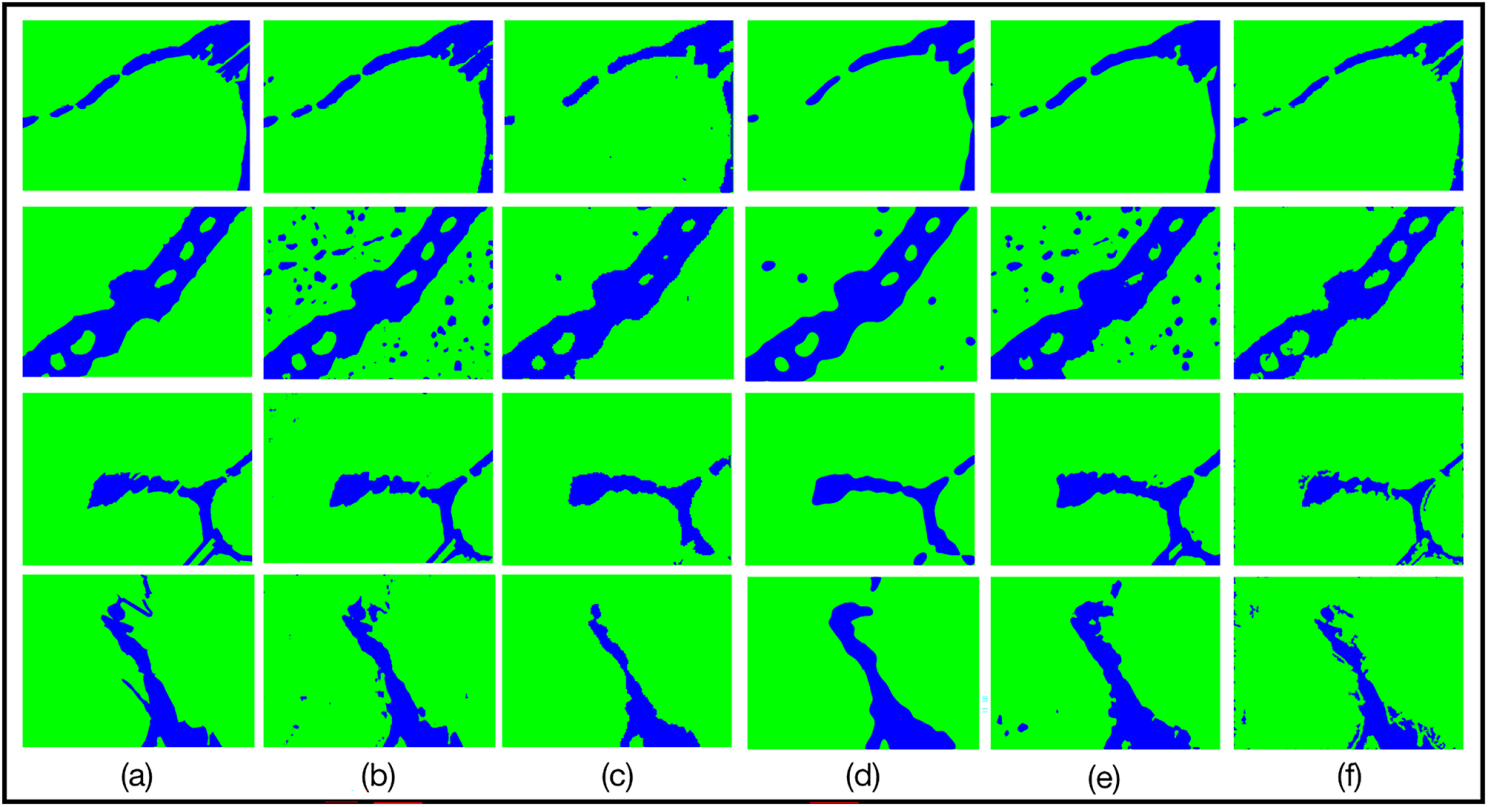
Comparison between the fully supervised deep learning methods and our CES method. (**a**) ground truth; (**b**) FCN method; (**c**) SegNet method; (**d**) PSPNet method; (**e**) Unet++ method; (**f**) CES (GBDT and post-processing process).

We also compare our method with deep learning methods including fully convolutional network (FCN)^22^, SegNet^23^, PSPNet^24^ and Unet++^25^. All of these deep learning methods use the annotation of whole images. The hyperparameters used for training FCN, SegNet, PSPNet and Unet++ are shown in Table S8. The results are shown in Fig. 5 and Table 2, S5, S6 and S7. Our method is about 10% lower on IoU and mIoU than the deep learning methods in the case of annotating only 0.2% pixels in each image. Since the deep learning methods are trained with full annotation and our method is based on a very small number of annotated pixels, the results with our method are lower than the ones with deep learning methods.

**Table 2:** The IoU (blue area), mIoU, Accuracy, Dice (blue area) and mDice of Image A1 using fully supervised deep learning methods and our CES method which is trained on group B.

Methods	IoU	mIoU	Accuracy	Dice	mDice
A1					
FCN	0.925	0.957	0.990	0.961	0.978
SegNet	0.748	0.855	0.966	0.856	0.919
PSPNet	0.763	0.864	0.968	0.866	0.924
Unet++	0.906	0.946	0.987	0.951	0.972
CES	0.788	0.878	0.972	0.881	0.933

### Segmentation for other data sets

**Figure 6:**
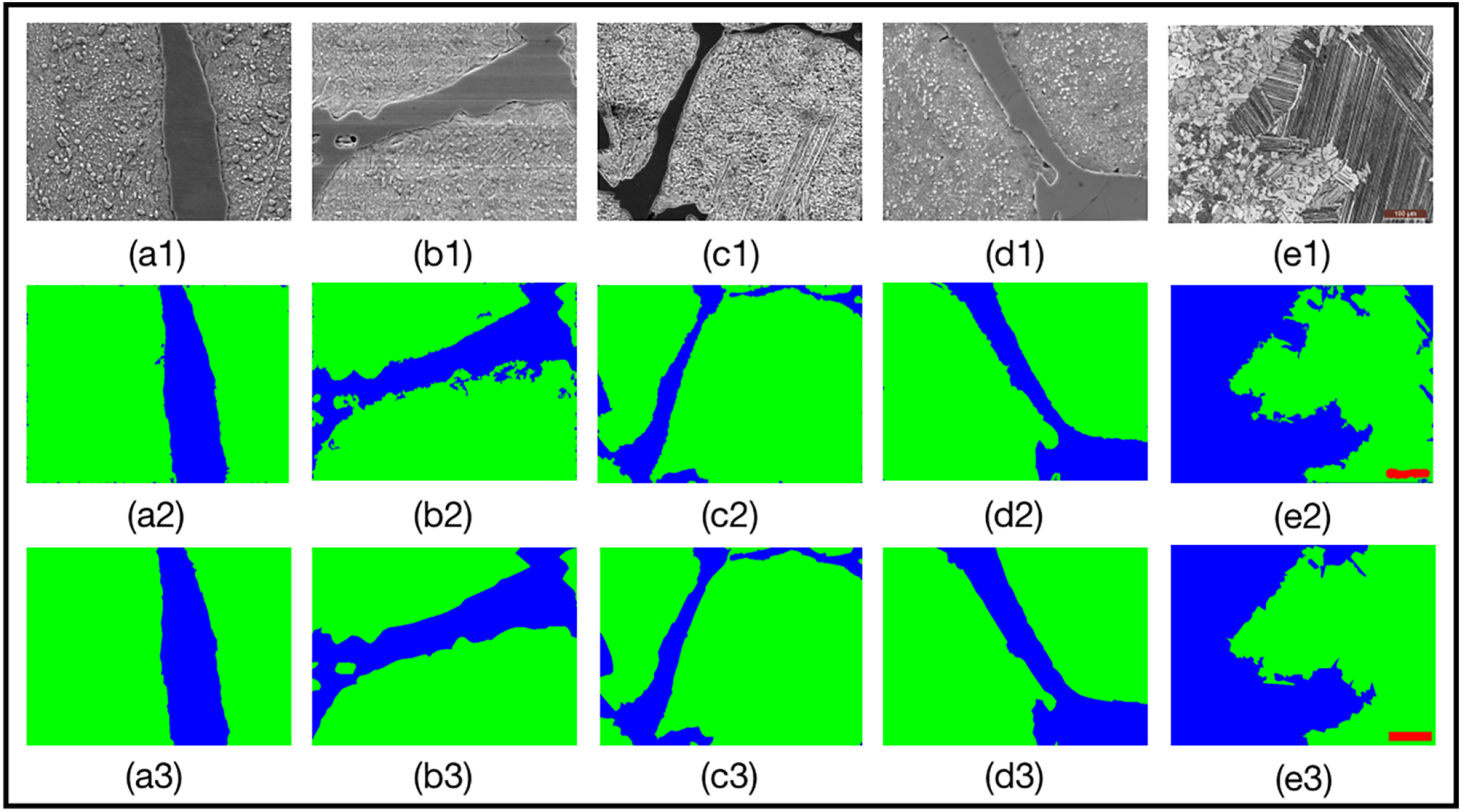
Results with our CES models after post-processing. (**a1**,**b1**,**c1**,**d1**) are the original carbon steel images; e1 is the original *TiAl* image; (**a2**,**b2**,**c2**,**d2**,**e2**) are the results with our method, respectively; (**a3**,**b3**,**c3**,**d3**,**e3**) are the ground truth of the images. The primary microconstituent of a1, b1 and d1 is spheroidite, and the primary microconstituent of c1 is pearlite and spheroidite. The primary microconstituent of e1 is equiaxed structure and lamellar structure.

The proposed method was also applied to several other images of material data sets, including other images from carbon steel images^8^, titanium alloy (*TiAl*) images, wood image data, Cross-sectional morphology of Pt-Al coating image data, Cross-sectional morphology of WC–Co coating image data and ceramics image data. The segmentation model of carbon steel images is trained on two images, mentioned in the above section, which are shown in Fig. 2, S2, and S3. We select the best model from the training model of each group in Fig. 1. For the *TiAl* data set including five images, a segmentation model is trained with two training images and tested on other three testing images. The training process is shown in Figure S7. For the rest data sets, we train and test on two images of each data set. The final models are adopted to segment other images in the same data sets for validation. Moreover, some results contain small isolated regions, so we use flood fill algorithm^26^ to post-process the results and obtain the final segmentation results. The post-processed segmentation results are shown in Fig. 6 and Figure S8. Table 3 describes the post-processed segmentation effect of images in Fig. 6 with an average mIoU of 0.89. Further, the results of other 5 data sets are shown in Figure S9 and Table S9. Our method performs well on most boundaries, and the average mIoU reaches 0.79.

**Table 3:** The mIoU, Accuracy and mDice with our CES method on carbon steel images^8^, and titanium alloy (*TiAl*) images in Fig. 6. a1, b1, c1, d1, e1 are corresponding with same indices in Fig. 6.

Index	a1	b1	c1	d1	e1
mIoU	0.942	0.885	0.894	0.899	0.860
Accuracy	0.982	0.954	0.973	0.967	0.956
mDice	0.970	0.938	0.942	0.946	0.925

## Discussion

In this work, we propose the “center-environment segmentation” (CES) method based on environment features and the annotation input of domain knowledge, which makes full use of the pixel’s information for material image segmentation. Our method is verified on 6 data sets, containing carbon steel images, wood images, Cross-sectional morphology of *Pt-Al* coating images*,* Cross-sectional morphology of *WC–Co* coating images, ceramics images and *TiAl* images. These data sets cover images with different textures, different luminance and different number of categories, demonstrating the robustness of our method for different images. We evaluate 6 carbon steel images in detail and compare with 18 typical methods, including 3 non-learning methods, 11 machine learning methods and 4 deep learning methods, which comprehensively verifies the performance of our method. Compared with non-learning methods and machine learning methods, our method performs better (Even though the watershed algorithm performs better than our method on carbon steel images in Fig. 1, it leads to more than 10% downgrade on IoU of foreground and mIoU as shown in Figure S10 and Table S10, S11, S12 and S13). This is because that most of non-learning methods only consider the numerical information of a single pixel, neglecting the relationship among neighborhood pixels in ROI. While our method combines 4 kinds of effective environment features (Gabor filter, Hu moment, HoG, GLCM) by aggregating neighborhood pixels’ feature of the center pixel in the ROI, which enhances the ability of feature expression. Then, we use supervised learning algorithms to learn the intrinsic rules of combination feature from the center-environment of each pixel. Compared with supervised learning algorithms, since GBDT performs best in many classifiers, our method selects GBDT as the final classifier (The average mIoU is 0.844 on A1, A2, C1 and C2 of Fig. 1 as shown in Table 1, S2, S3 and S4). Compared with unsupervised methods, e.g., Han’s method^11^ and K-means method^16^, the segmentation results with our method is better due to the sufficient environment feature information of pixel’s ROI. Deep learning methods (FCN, SegNet, PSPNet, Unet++) can also segment the material images, while complete and massive annotations are needed to train the model. With the rapid development of deep learning, the work based on deep learning methods is necessary in the future. In the current work, we propose the “center-environment segmentation” method based on the annotation input of domain knowledge to solve the problem of small sample with less annotation cost. Furthermore, small isolated regions in the segmented images affect the final results, so we remove them to improve the segmentation accuracy, as shown in Figure S8 in supplemental information file.

This work acquires the center-environment features of pixels based on annotation inputs with domain knowledge in the training process. In other words, the training process is achieved by predefining the number of phase categories, annotation, extraction of features, and training the segmentation model. When some regions are incorrectly classified, with the expert’s domain knowledge, we can focus on extracting center-environment feature from the specific regions and train them again. For instance, for two training samples from carbon steel image set in Fig. 2, the mIoU is improved from 0.737 to 0.748 by the second training round, as shown in Fig. 3. In the following training rounds, the mIoU is gradually increased. Although the unsupervised learning methods, e.g., K-means method, can be executed without the annotation, the mIoU is only 0.648, which is much lower than the supervised learning methods, as shown in Table 1, S2, S3 and S4. The reason is that with our proposed model, the supervised learning methods is able to modify the incorrectly segmented regions of the previously training results with domain knowledge annotation inputs.

Although the proposed method achieves well segmentation results in the experiments, it still has some drawbacks and deserves further improvement. In the experiment of the carbon steel image segmentation, we terminate the training in the sixth round. However, for different types of images, the optimal number of training rounds is different. Therefore, it is necessary to design a suitable strategy to adaptively terminate the training iteration. Moreover, the segmentation accuracy of the proposed method need to be further improved. We hope to achieve a trade-off between the annotation cost and segmentation accuracy in deep learning methods to obtain better segmentation results in a wide range of material images.

## Methods

**Figure 7:**
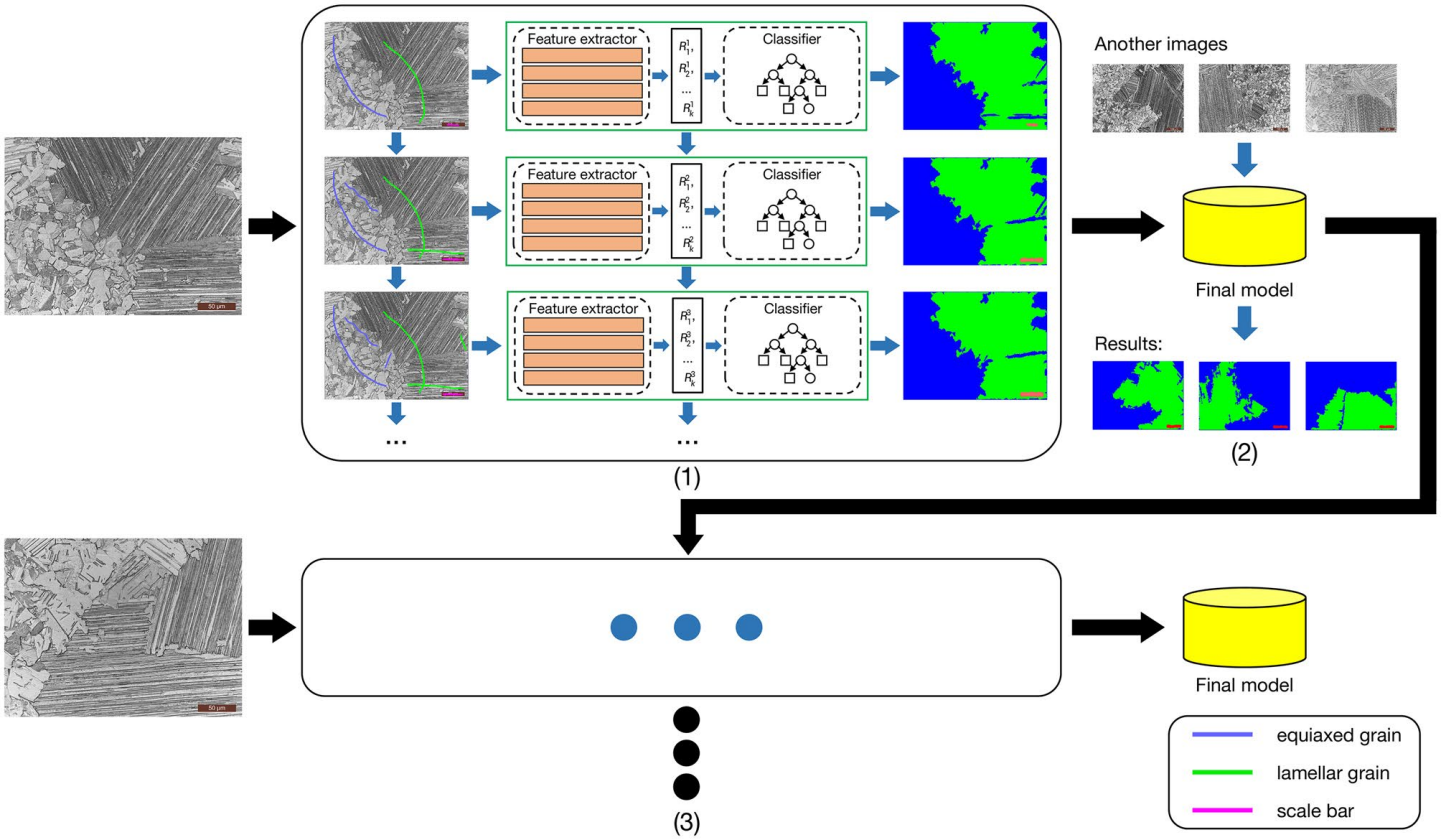
The flow chart of our CES method. (1) Training process of per round. $R_{1}^{i} ,R_{2}^{i} ,...R_{k}^{i}$ are the feature vector sets including all features of *i* rounds on the selected pixels. (2) Using the final model generated in training process to segment other images of the same data set. (3) Training model by using another image again if the model is not good enough.

Figure 7 illustrates the flow chart of the segmentation method. The main process is described as follows: (1) in preprocessing stage, the ROI is represented by a patch with $n * n$ pixels and the phase category numbers $k$ is determined according to expert domain knowledge of the material; (2) $k$ curves in different colors representing $k$ phases in $k$ regions of an input sample image are drawn based on domain knowledge; (3) pixels on the curves and pixels in the ROI of the selected pixels are put into feature extractors, which are described in the supplemental information, to generate feature vector set $R = (R_{{1}}^{i} ,R_{2}^{i} ,...,R_{k}^{i} )$ where $R_{*}^{i}$ represents the feature vector for all features of *i* rounds on the selected pixels in ${*}$ regions; (4) the segmentation model is trained on the feature vector set $R$. The steps from (1) to (4) are one training round. If some regions are segmented wrongly, the steps (2), (3) and (4) are particularly repeated on the wrongly segmented areas as one more round. The extracted feature vectors of pixels in new round are added into the previous feature vector set $R = (R_{{1}}^{i} ,R_{2}^{i} ,...,R_{k}^{i} )$ which is used to further train the segmentation model. The segmentation model can be further trained with other image samples if the model is not good enough. The final model can be used to segment other data in the same set.

The center-environment feature of each pixel, i.e., the feature vector of each pixel, is constituent of four different types of the texture features: Gabor filter, Hu moment, HoG, and GLCM. The construction of center-environment feature vectors are described in detail in the supplementary information file. Many machine leaning methods can be used to train the feature vector sets. For supervised machine learning methods, the curves drawn with domain knowledge are needed. On the other hand, if we adopt the unsupervised machine learning methods, the step of drawing curves can be saved. For instance, only $k$ clustering centers are set if K-means method is used to segment the material images. Experiments validate that GBDT performs the relative optimal results among the compared algorithms, whose mIoU is 0.787, as shown in Fig. 4 and Table 1, S2, S3 and S4. Thus, we choose the GBDT algorithm as classifier for material images. The parameters of the GBDT algorithm are provided in Table S14.

## Supplementary Information

Supplementary Information.

## Data Availability

The datasets generated during and/or analysed during the current study are available from the corresponding author on reasonable request. All data generated or analysed during this study are included in this published article (and its Supplementary Information file).
