# Supplementary material for: Center-environment feature models for materials image segmentation based on machine learning
Source: Sci Rep. 2022 Jul 28;12:12960. doi: 10.1038/s41598-022-16824-w (PMC9334618; doi:10.1038/s41598-022-16824-w)
Supplement: Supplementary file 1 — Supplementary Information. [file 41598_2022_16824_MOESM1_ESM.pdf]

Supplementary Information file for Center-environment feature models for materials image segmentation based on machine learning

Yuexing Han[1,*], Ruiqi Li[1], Shen Yang[1], Qiaochuan Chen[1], Bing Wang[1], Yi Liu[2]

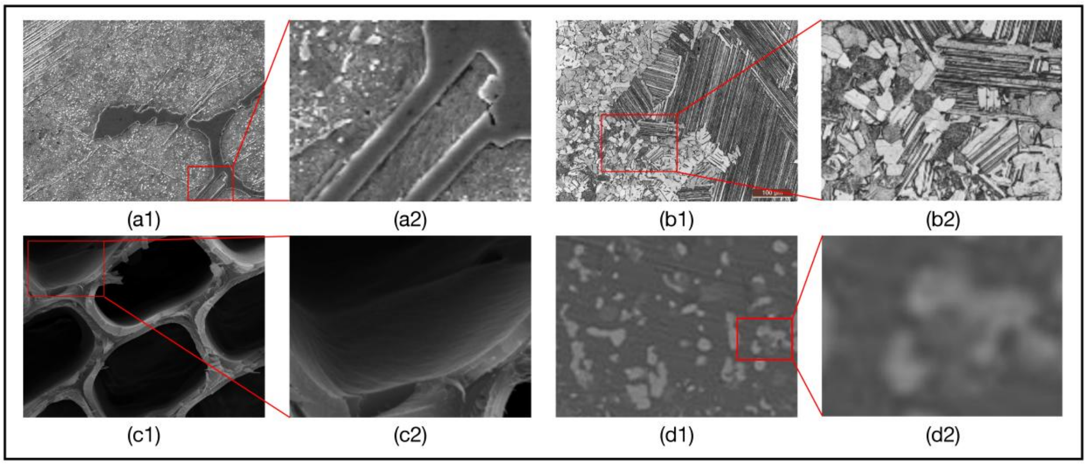

Figure S1. Some examples of fuzzy boundaries and complicated irregular texture in different kinds of images. a1 is a carbon steel image; b1 is a *TiAl* image; c1 is a wood image; and d1 is a Cross-sectional morphology of *Pt-Al* coating image.

Table S1. The IoU and mIoU of each groups by using the final model.

Model \ IoU/mIoU	A1	A2	B1	B2	C1	C2
Group A	/	/	0.405/ 0.675	0.751/ 0.859	0.637/ 0.800	0.648/ 0.803
**Group B**	0.788/ 0.878	0.846/ 0.898	/	/	0.663/ 0.813	0.623/ 0.785
Group C	0.819/ 0.896	0.834/ 0.889	0.510/ 0.729	0.660/ 0.799	/	/

Table S2. The IoU (blue area), mIoU, Accuracy, Dice (blue area) and mDice of Image A2 using other methods and our CES method trained on group B.

A2 / Methods	IoU	mIoU	Accuracy	Dice	mDice
MRF	0.674	0.781	0.908	0.806	0.873
Watershed	**0.959**	**0.973**	**0.990**	**0.979**	**0.986**
Han	0.677	0.777	0.903	0.808	0.871
Meanshift	0.544	0.658	0.821	0.705	0.788
DT	0.590	0.697	0.847	0.742	0.816
KNN	0.644	0.757	0.894	0.783	0.857
K-means	0.298	0.551	0.820	0.459	0.675
Navie Bayes	0.672	0.780	0.909	0.804	0.872
SVM(RBF)	0.696	0.795	0.915	0.821	0.883
SVM(Sigmoid)	0.625	0.731	0.872	0.769	0.840
RF	0.694	0.792	0.912	0.819	0.881
AdaBoost	0.723	0.811	0.920	0.839	0.893
XGBoost	0.752	0.830	0.928	0.858	0.905
DART	0.742	0.825	0.927	0.852	0.902
GBDT	0.762	0.837	0.932	0.865	0.910
**CES**	0.846	0.898	0.960	0.917	0.945

Table S3. The IoU (blue area), mIoU, Accuracy, Dice (blue area) and mDice of Image C1 using other methods and our CES method trained on group B.

C1 / Methods	IoU	mIoU	Accuracy	Dice	mDice
MRF	0.124	0.226	0.386	0.221	0.357
Watershed	**0.825**	**0.903**	**0.982**	**0.904**	**0.947**
Han	0.156	0.433	0.725	0.270	0.550
Meanshift	0.407	0.646	0.894	0.578	0.759
DT	0.386	0.634	0.890	0.557	0.747
KNN	0.425	0.671	0.921	0.597	0.776
K-means	0.484	0.715	0.948	0.652	0.812
Navie Bayes	0.351	0.622	0.898	0.519	0.731
SVM(RBF)	0.486	0.711	0.939	0.654	0.810
SVM(Sigmoid)	0.245	0.496	0.765	0.394	0.624
RF	0.489	0.710	0.934	0.657	0.810
AdaBoost	0.501	0.717	0.938	0.668	0.817
XGBoost	0.550	0.745	0.943	0.710	0.839
DART	0.551	0.746	0.945	0.711	0.840
GBDT	0.569	0.756	0.948	0.725	0.848
**CES**	0.663	0.813	0.965	0.797	0.889

Table S4. The IoU (blue area), mIoU, Accuracy, Dice (blue area) and mDice of Image C2 using other methods and our CES method trained on group B.

Methods \ C2	IoU	mIoU	Accuracy	Dice	mDice
MRF	0.133	0.193	0.330	0.235	0.319
Watershed	**0.786**	**0.878**	**0.973**	**0.880**	**0.933**
Han	0.119	0.164	0.285	0.213	0.279
Meanshift	0.017	0.453	0.889	0.034	0.488
DT	0.298	0.542	0.803	0.459	0.669
KNN	0.360	0.603	0.859	0.530	0.723
K-means	0.476	0.693	0.917	0.645	0.799
Navie Bayes	0.251	0.500	0.768	0.401	0.629
SVM(RBF)	0.395	0.631	0.877	0.567	0.747
SVM(Sigmoid)	0.153	0.279	0.462	0.265	0.420
RF	0.362	0.604	0.859	0.532	0.724
AdaBoost	0.313	0.560	0.823	0.477	0.685
XGBoost	0.429	0.656	0.892	0.600	0.769
DART	0.450	0.671	0.901	0.620	0.782
GBDT	0.471	0.686	0.909	0.640	0.794
**CES**	0.623	0.785	0.951	0.767	0.870

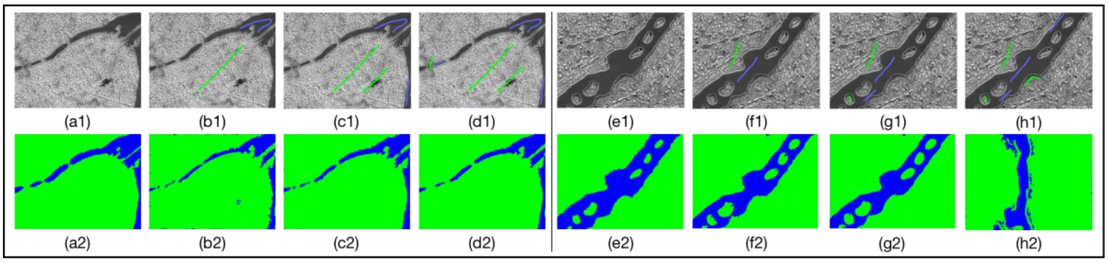

Figure S2. Training process of images in group A. (a1, e1) are the original images of group A. (a2, e2) are the ground truth. (b1, c1, d1) and (f1, g1, h1) are the training process for a1 and e1, respectively. (b2, c2, d2) and (f2, g2, h2) are the results corresponding with per training round.

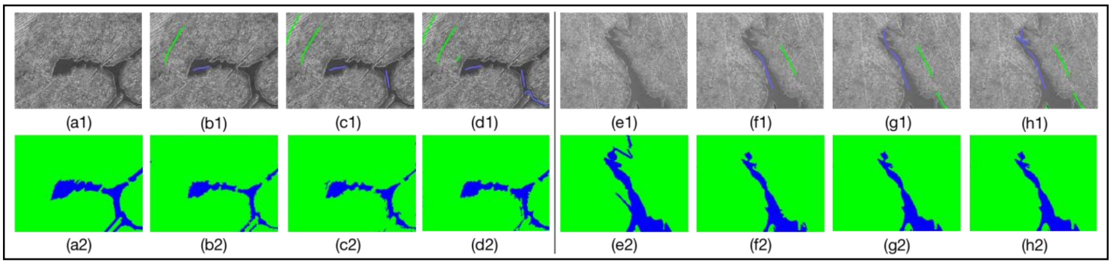

Figure S3. Training process of images in group C. (a1, e1) are the original images of group C. (a2, e2) are the ground truth. (b1, c1, d1) and (f1, g1, h1) are the training process of the two images. (b2, c2, d2) and (f2, g2, h2) are the results corresponding with per training round.

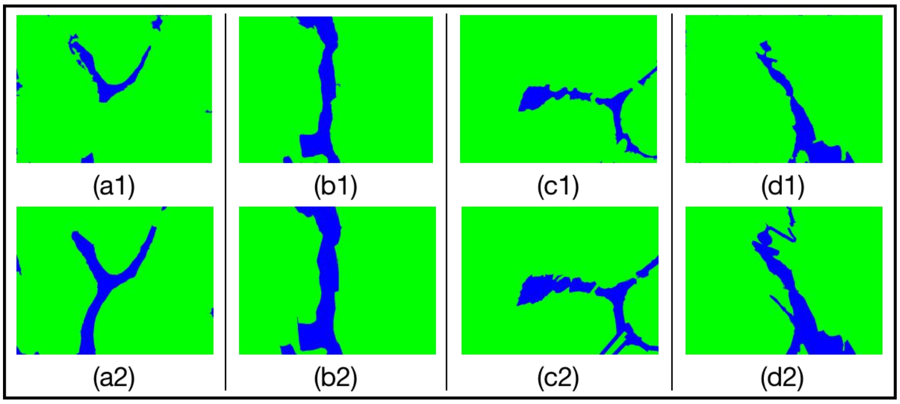

Figure S4. Results with the model generated by group A. (a1, b1, c1, d1) are the segmentation results corresponding to the original images (B1, B2, C1, C2), respectively. (a2, b2, c2, d2) are the ground truth of them.

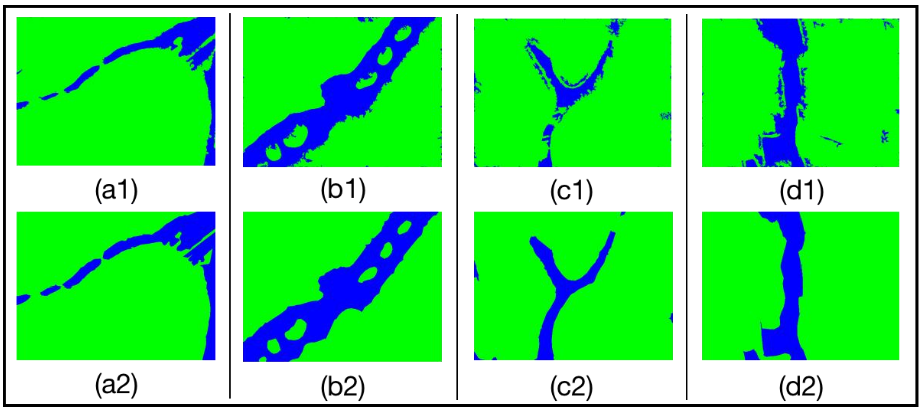

Figure S5. Results with the model generated by group C. (a1, b1, c1, d1) are the segmentation result corresponding to the original images (A1, A2, B1, B2), respectively. (a2, b2, c2, d2) are the ground truth of them.

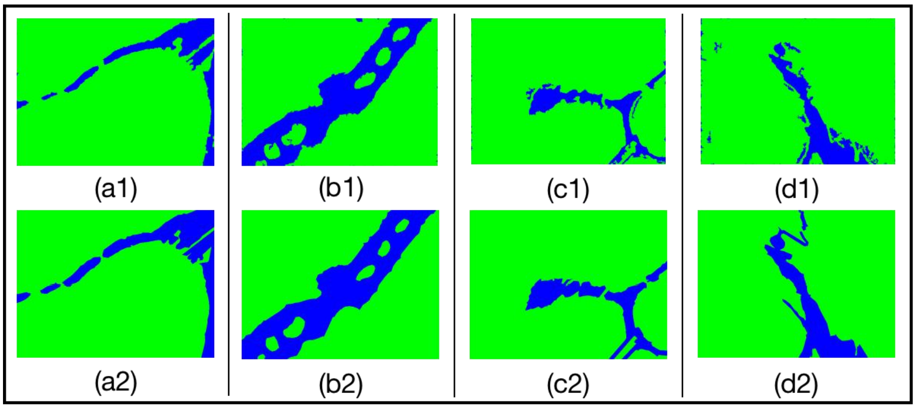

Figure S6. Results with the model generated by group B. (a1, b1, c1, d1) are the segmentation result corresponding to the original images (A1, A2, C1, C2), respectively. (a2, b2, c2, d2) are the ground truth of them.

Table S5. The IoU (blue area), mIoU, Accuracy, Dice (blue area) and mDice of Image A2 using fully supervised deep learning methods and our CES method which is trained on group B

A2 / Methods	IoU	mIoU	Accuracy	Dice	mDice
FCN	0.808	0.864	0.940	0.894	0.926
SegNet	0.899	0.931	0.972	0.947	0.964
PSPNet	**0.907**	**0.937**	**0.975**	**0.951**	**0.967**
Unet++	0.796	0.855	0.936	0.886	0.921
**CES**	0.846	0.898	0.960	0.917	0.945

Table S6. The IoU (blue area), mIoU, Accuracy, Dice (blue area) and mDice of Image C1 using fully supervised deep learning methods and our CES method which is trained on group B

C1 / Methods	IoU	mIoU	Accuracy	Dice	mDice
FCN	**0.894**	**0.941**	**0.990**	**0.944**	**0.969**
SegNet	0.764	0.869	0.976	0.866	0.927
PSPNet	0.772	0.874	0.977	0.872	0.929
Unet++	0.792	0.882	0.976	0.884	0.935
**CES**	0.663	0.813	0.965	0.797	0.889

Table S7. The IoU (blue area), mIoU, Accuracy, Dice (blue area) and mDice of Image C2 using fully supervised deep learning methods and our CES method which is trained on group B

C2 / Methods	IoU	mIoU	Accuracy	Dice	mDice
FCN	**0.828**	**0.903**	**0.979**	**0.906**	**0.947**
SegNet	0.725	0.846	0.970	0.841	0.912
PSPNet	0.737	0.849	0.964	0.848	0.914
Unet++	0.703	0.828	0.957	0.826	0.901
**CES**	0.623	0.785	0.951	0.767	0.870

Table S8. The hyperparameters of FCN, SegNet, PSPNet and Unet++ in our experiments. Adam indicates Adaptive Moment Estimation.

parameter / Methods	Epoch	Learning rate	Optimizer	Batch size
FCN	30	1e-3	Adam	1
SegNet	50	1e-3	Adam	1
PSPNet	30	1e-3	Adam	1
Unet++	30	1e-3	Adam	1

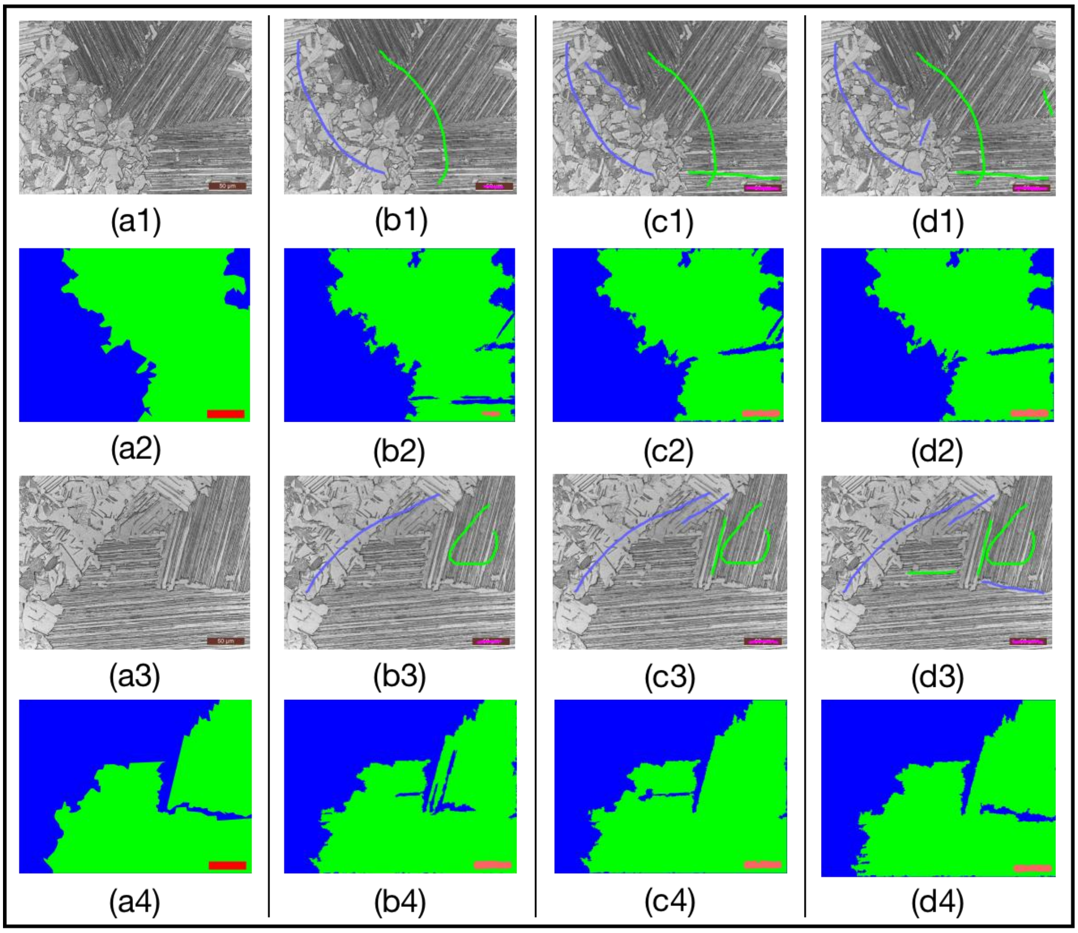

Figure S7. Training process of segmentation model on *TiAl* image data set. (a1, a3) are the original images; (a2, a4) are the ground truth, respectively. (b1, c1, d1) and (b3, c3, d3) show the training process, respectively. (b2, c2, d2) and (b4, c4, d4) are the results corresponding with (b1, c1, d1) and (b3, c3, d3), respectively.

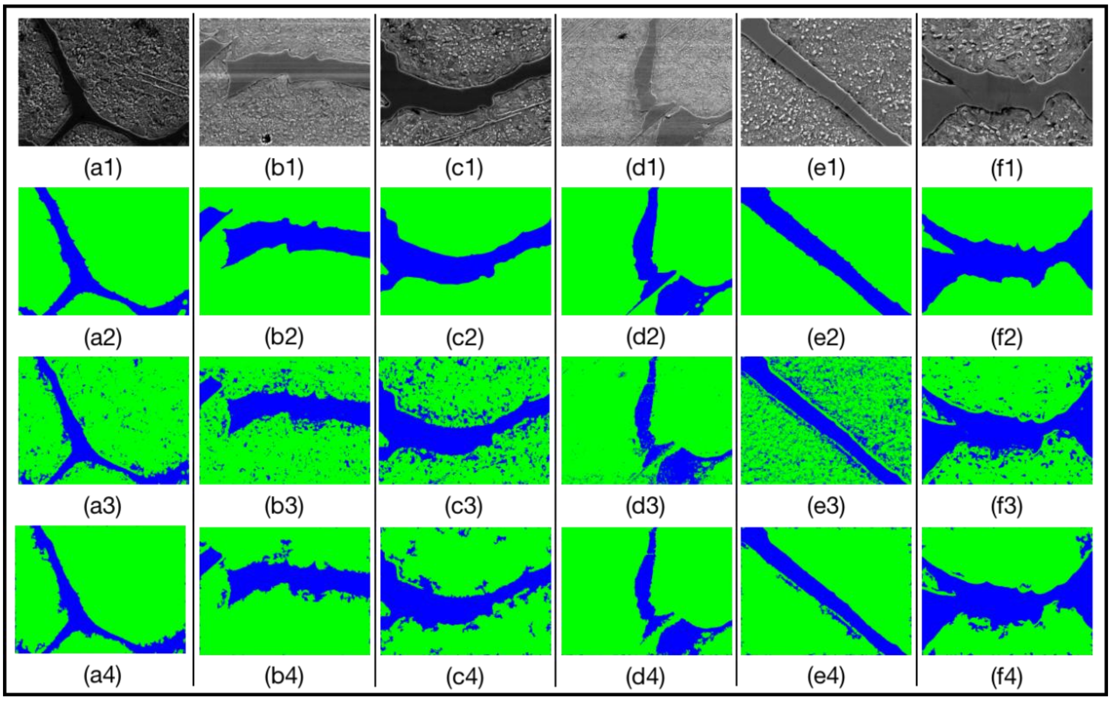

(1)

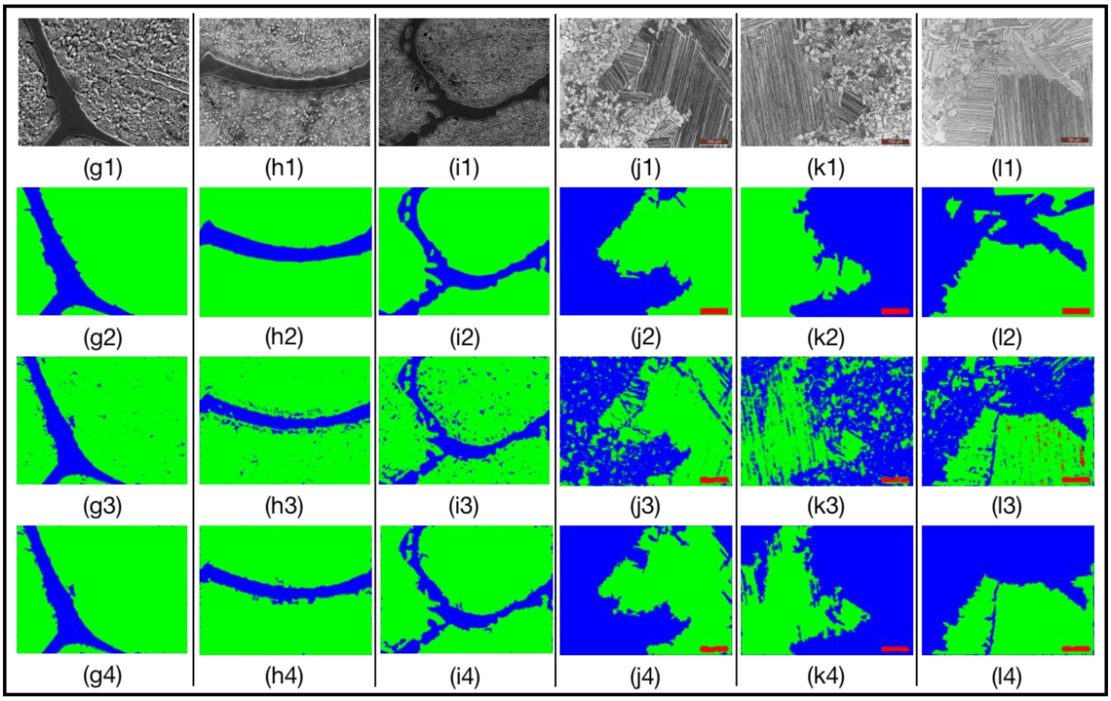

(2)

Figure S8. Results of using the final model in training process on multiple data sets before and after post-processing. (a1, b1, c1, d1, e1, f1, g1, h1, i1) are the original

carbon steel images. (j1, k1, l1) are the original *TiAl* images. (a2, b2, c2, d2, e2, f2, g2, h2, i2, j2, k2, l2) are the ground truth. (a3, b3, c3, d3, e3, f3, g3, h3, i3, j3, k3, l3) are the results before post-processing. (a4, b4, c4, d4, e4, f4, g4, h4, i4, j4, k4, l4) are the results after post-processing.

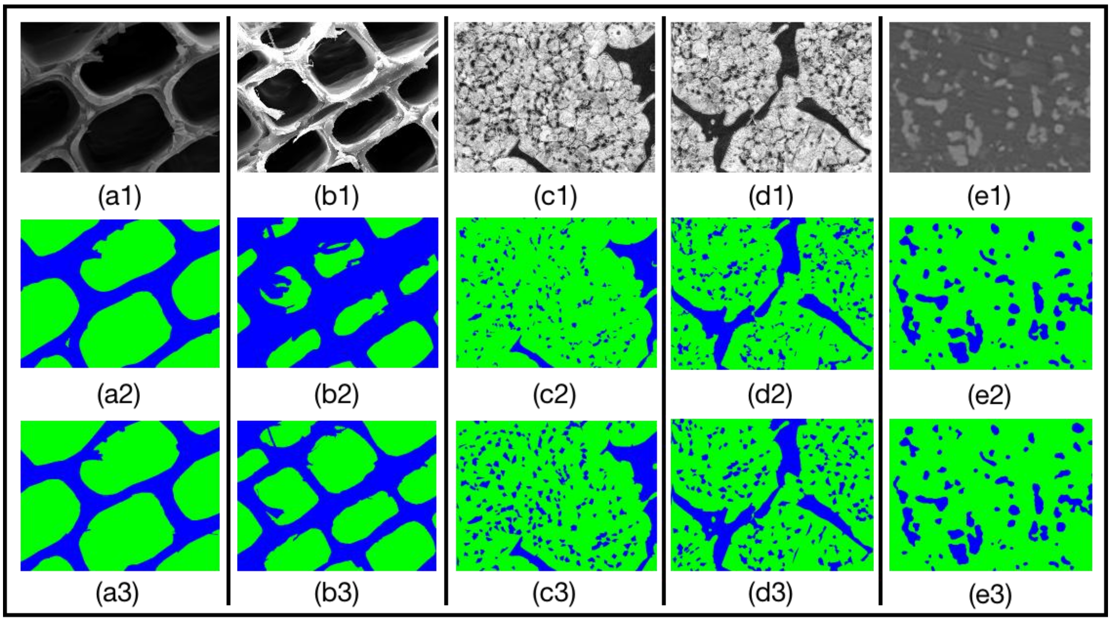

(1)

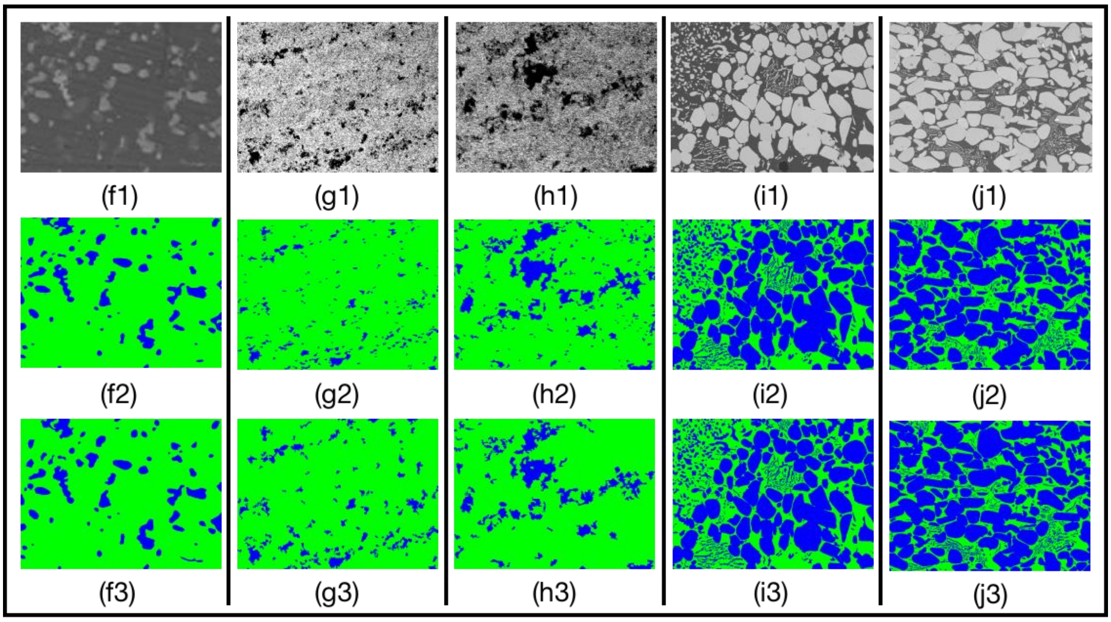

(2)

Figure S9. Results of other data set. (a1, b1) are the original images of wood. (a2, b2) are the results. (a3, b3) are the ground truth. (c1, d1) are another kind of original images from carbon steel data set. (c2, d2) are the results. (c3, d3) are the ground truth. (e1, f1) are the original images of Cross-sectional morphology of *Pt-Al* coating. (e2, f2) are the results. (e3, f3) are the ground truth. (g1, h1) are the original images of Cross-sectional morphology of *WC-Co* coating. (g2, h2) are the results. (g3, h3) are the ground truth. (i1, j1) are the original images of ceramics. (i2, j2) are the results. (i3, j3) are the ground truth.

Table S9. The average IoU (blue area), mIoU, Accuracy, Dice (blue area) and mDice with the proposed segmentation method on the sets of Wood, Carbon Steel, *Pt-Al*, *Wc-Co*, and Ceramics images.

Data set	Wood	Carbon Steel	*Pt-Al*	*Wc-Co*	Ceramics
IoU	0.710	0.635	0.783	0.542	0.825
mIoU	0.711	0.769	0.875	0.743	0.857
Accuracy	0.826	0.917	0.971	0.947	0.927
Dice	0.824	0.775	0.873	0.694	0.904
mDice	0.820	0.862	0.928	0.832	0.922

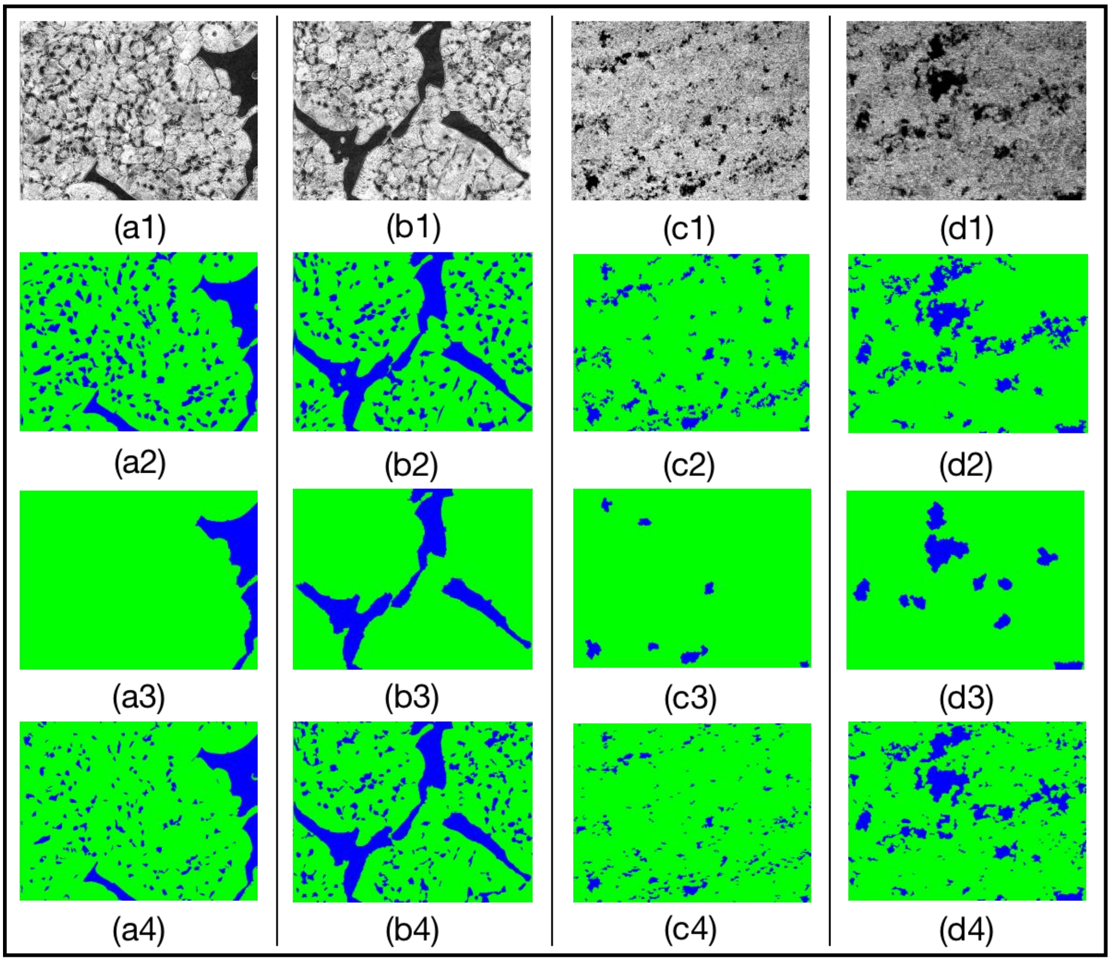

Figure S10. Comparison between the watershed algorithm and our method on different data set. (a1, b1) are another kind of original images from carbon steel data set. (a2, b2) are the ground truth. (a3, b3) are the results of the watershed algorithm. (a4, b4) are the results of our method. (c1, d1) are the original images of Cross-sectional morphology of *WC-Co* coating. (c2, d2) are the ground truth. (c3, d3) are the results of the watershed algorithm. (c4, d4) are the results of our method.

Table S10. The IoU (blue area), mIoU, Accuracy, Dice (blue area) and mDice of Image a1 with the proposed segmentation method and the watershed algorithm on images in Figure S10.

a1 / Methods	IoU	mIoU	Accuracy	Dice	mDice
Watershed	0.363	0.615	0.876	0.533	0.726
**CES**	**0.570**	**0.735**	**0.912**	**0.731**	**0.837**

Table S11. The IoU (blue area), mIoU, Accuracy, Dice (blue area) and mDice of Image b1 with the proposed segmentation method and the watershed algorithm on images in Figure S10.

b1 / Methods	IoU	mIoU	Accuracy	Dice	mDice
Watershed	0.545	0.711	0.892	0.705	0.819
**CES**	**0.700**	**0.802**	**0.921**	**0.824**	**0.887**

Table S12. The IoU (blue area), mIoU, Accuracy, Dice (blue area) and mDice of Image c1 with the proposed segmentation method and the watershed algorithm on images in Figure S10.

c1 / Methods	IoU	mIoU	Accuracy	Dice	mDice
Watershed	0.201	0.567	0.935	0.335	0.650
**CES**	**0.411**	**0.677**	**0.945**	**0.583**	**0.777**

Table S13. The IoU (blue area), mIoU, Accuracy, Dice (blue area) and mDice of Image d1 with the proposed segmentation method and the watershed algorithm on images in Figure S10.

d1 / Methods	IoU	mIoU	Accuracy	Dice	mDice
Watershed	0.401	0.660	0.924	0.572	0.765
**CES**	**0.673**	**0.809**	**0.950**	**0.804**	**0.888**

Table S14. The training parameters of GBDT in our experiments.

Parameters / Method	num_ leaves	learning _rate	num_ trees	feature_ fraction	bagging _fraction	bagging _freq
GBDT	93	0.05	300	0.9	0.8	5

Introduction of Feature extractor

Feature extraction is the first step for the image segmentation. In general, the material images contain rich and complex texture information. The feature vectors are extracted to describe the texture information. Here, the feature extractor is designed to extract four types of the texture features: Gabor filter, Hu moment, HoG, and GLCM, and combine them into center-environment features. The ROI of a pixel and its texture features are shown in Figure S11.

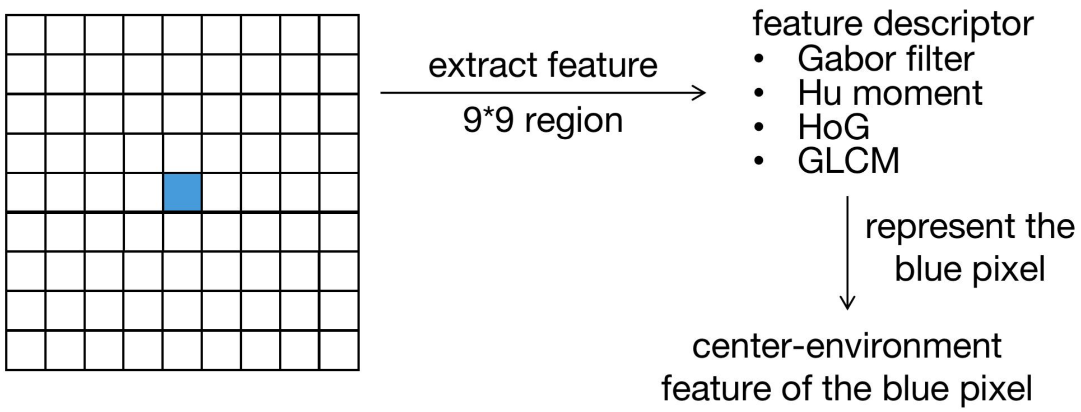

Figure S11. The ROI of a pixel and its texture features.

Gabor filter is a linear feature extractor for extracting edge information. The Gabor filters can obtain the responses of an image in different frequencies. Then, the features can be extracted from different frequencies. Since 2-D Gabor filters can well reflect the edge information in 2-D image, we use 2-D Gabor filters in six directions (0°, 30°, 60°, 90°, 120°, 150°), i.e., six Gabor filters, to convolute the original image to obtain the 6-dimensional feature vector for each pixel. Here, Gabor features are denoted as f_{ga} .

Hu moment is the shape-based description features with translation invariance, rotation invariance and scale invariance. Hu moment is composed of seven invariant moments which can quickly reflect the image contour information. In our method,

7-D features are calculated with the Hu moment for each pixel in ROI, denoted as f_{hu}.

HoG describes the gradient direction of pixels and reflects the distribution characteristics of gradient direction and intensity of edge in the image. Here, the number of histograms is set to 9. Each histogram records the number of changes in the gradient direction of a pixel in ROI, and a total of 9-D features of each pixel are obtained, denoted as f_{hg}.

GLCM shows the distribution of co-occurrence pixels, which can reflect the characteristics of image texture in the aspects of direction, distance and transformation. Here, $G_{\varepsilon-a}$ is used to represent a GLCM that is calculated to determine the image features. ε and a means the distance and direction of each pixel pair, respectively. In the paper, ε is set to 2, and a is set to (0°, 45°, 90°, 135°), described as $(G_{2\text{-}0^\circ}, G_{2-45^\circ}, G_{2-90^\circ}, G_{2-135^\circ})$. Then, four features, named as contrast $Con_{\varepsilon-a}$, angular second moment $Asm_{\varepsilon-a}$, information entropy $Ent_{\varepsilon-a}$ and correlation degree $Corr_{\varepsilon-a}$ are calculated from the $G_{\varepsilon-a}$ in the ROI of a target pixel. Therefore, we obtain 16-D GLCM features of each pixel. In order to reduce the dimension of features, we calculate the variance of four features for four directions, denoted as $(\text{var}_{Con}, \text{var}_{Asm}, \text{var}_{Ent}\ \text{var}_{Corr})$. Finally, we obtain 4-D features of each pixel as GLCM features, denoted as f_{gm}.

In this way, pixel i can be represented by 6 Gabor features, 7 Hu moment features, 9 HoG features and 4 GLCM features, and the feature vectors u_i can be formulated as:

$$u_i = F(f_{ga}^i, f_{hu}^i, f_{hg}^i, f_{gm}^i), \tag{1}$$

where $F(\cdot)$ is a feature extractor to aggregate these 4 types of the features to 26-D features.

Once category number k is determined with the expert domain knowledge, we draw k curves to extract partial pixels in k different types of the regions. For each pixel covered by the drawn curves, we extract pixels in a patch of $n*n$ size as ROI and put them into feature extractor described in Formula (1). Thus, the feature vectors of partial pixels in each region are generated and aggregated as feature vector set R_i. Then, the feature vector set is used to train the segmentation model. The feature vector set R_i can be represented as:

$$R_i = C(u_{1,}u_{2,}...,u_m), \quad (2)$$

where $C(\cdot)$ merges feature vectors of pixels in the i region. The feature vectors of the rest pixels are also generated by the feature extractor for classification. The classification results of all pixels are obtained with the trained segmentation model. When some pixels are classified incorrectly, partial wrongly classified pixels are extracted with human-machine interaction and added into previous feature vector set R_i for further training of the segmentation model. Then, the classifier focuses on the newly added feature vectors and ensure misclassified pixels can be corrected.
